# Sick Leave Due to Stress, What are the Costs for Dutch Employers?

**DOI:** 10.1007/s10926-022-10042-x

**Published:** 2022-05-16

**Authors:** Sjors Wolvetang, Johanna Maria van Dongen, Erwin Speklé, Pieter Coenen, Frederieke Schaafsma

**Affiliations:** 1 Department of Public and Occupational Health, Amsterdam Public Health Research Institute, Amsterdam UMC, Vrije Universiteit Amsterdam, Amsterdam, The Netherlands; 2 Faculty of Science, Health Economics and Health Technology Assessment, Vrije Universiteit Amsterdam, Amsterdam, The Netherlands; 3 Arbo Unie, Occupational Health Service, Utrecht, The Netherlands

**Keywords:** Sick leave, Employer health costs, Cost of illness, Sick days, Burn-out

## Abstract

*Purpose* Stress-related illnesses are prevalent in Western society, causing sick leave and putting a heavy economic burden on employers and society. For Dutch employers it is particularly relevant to have insight into the costs of absenteeism due to stress-related illness, as they are legally obligated to continue payment of wages. Therefore, this study assessed the duration and costs of an episode of sick leave due to stress-related illness for Dutch employers. *Methods* Data on sick leave due to various stress-related illnesses were obtained from a nationwide occupational health service database. Stress-related illnesses included tension complaints, burn-out, overexertion, and other reactions to stress. The duration per sick leave episode was estimated in working days, after which the average cost per sick leave period was estimated using age- and gender-specific price weights. *Results* During the study period, 16,676 employees took 17,338 episodes of sick leave due to stress-related illness. On average, one episode of sick leave lasted 101 working days, for which the costs for the employer were on average €19,151 per worker. Women were responsible for most episodes of sick leave and were on average 37 days more absent per episode compared to men. Moreover, of all kinds of stress-related illnesses, burn-out had the longest duration of sick leave with 313 calendar days and 163 working days, resulting in an average cost of €30,770. *Conclusions* Sick leave due to stress-related illness places a heavy burden on employers and thus society. Further research should be conducted on how to reduce this burden.

## Introduction

In Europe, there has been an increasing trend in absenteeism due to illness over the past decade. The point prevalence of the number of individuals on sick leave increased from about 3 million to more than 4 million during this period [1]. This increasing trend is also visible in the Netherlands, where 4.3% of all working days were lost due to absenteeism in 2020 and the prevalence of sick leave is believed to have increased further because of Covid-19 [2]. The causes of sick leave vary widely, but it is known that mental illness is a major culprit [3]. In fact, the share of sick leave due to mental illness has grown to 42% of all sick leave days in the Netherlands in 2019 [4]. Stress-related illnesses, such as burn-out, overexertion, and tension complaints, are responsible for a large share of sick leave. An annual national working conditions survey showed that in 2019 about 1.3 million employees suffered from burnout complaints and that 37% of the employees on sick leave indicated stress and workload as the main reason for their absence [5].

Not only the Netherlands is plagued by stress-related mental disorders. Despite possible differences in diagnoses across countries, it is clear that stress-related disorders are a global problem with a large economic burden. For instance, a study showed that reduced nationwide productivity due to burn-out costs Germany €9 billion per year [6]. In the United States, reduced productivity due to stress-related absenteeism of physicians alone costs $4.6 billion per year [7]. Moreover, the total cost of stress-related illness were found to range from $221 million in Australia to $187 billion in the USA [8].

In the Netherlands, the economic burden of stress-related illnesses is largely carried by employers. In the event of sick leave, Dutch employers are obligated by law to continue to pay part of their employees’ wages for up to two years [9]. A more detailed description of the Dutch occupational health and social security system can be found in Box 1. The Dutch system is designed in such a way that employers experience a financial incentive to prevent employees from becoming ill due to work, and in the event of illness with sick leave that employers make an effort to support their employees’ return to work. This system, however, confronts employers with high costs, as they not only lose the productivity of the sick employee, but also have to continue to pay their wages.

Research among Dutch employers has shown that 34% of them are concerned about sick leave in their company [10]. The same research indicates that employers would like to gain more insight into the sick leave rates of their company and, in particular, the associated costs. Employers need this information to adequately prepare for absenteeism and to shield themselves from possible high related costs. Nevertheless, information regarding the duration and costs of sick leave due to stress-related illness is extremely scarce, despite the fact that such sick leave is highly prevalent. As far as we are aware, over the past years only few scientific studies estimated the duration of stress-related sick leave and, moreover, these have not been conducted in the Netherlands [11–13]. There is, however, a national working conditions survey that indicated that work-related stress cost Dutch employers €3.1 billion in 2018 [5]. This survey was based on self-reported complaints rather than on diagnoses made by a doctor and the costs and duration were not specified in any further detail. Therefore, this study aimed to assess with great precision and detail the duration and costs of an episode of sick leave due to stress-related illness for employers in the Netherlands in the period of 2015 up to and including 2019.

Box 1: The Dutch Occupational Health and Social Security System In the Netherlands, as soon as an employee becomes ill and/or is unable to work, he or she reports this to his or her employer. In the event of persistent illness, an employee will visit an occupational physician within six weeks of sick leave. Dutch employers are obligated by law to have a contract with an occupational physician who is typically employed by an occupational health service and provides advice regarding work ability and return to work of their sick employees [14]. These physicians determine the cause of the employee’s absenteeism through an anamnesis, conversations and, if possible, by means of information available from a (general) practitioner. Furthermore, they support the employee’s return to work process and inform the employer about the estimated duration of absenteeism. The employer and employee draw up an action plan for return to work and have regular progress meetings. Dutch employers are obligated to continue to pay at least 70% of the employee’s gross wages during the employee’s sick leave up to two years [9]. However, it is generally arranged in the collective labor agreement that the employer pays 100% of the gross wages in the first year and 70% in the second year [15]. Situations also arise where it has been agreed that the employee will receive 100% of his/her gross salary during the entire period of sick leave. If an employee remains on sick leave for more than two years and is therefore not yet able to work fully, the employer may dismiss the employee and is no longer responsible for payment of wages. From that moment on, the employee is entitled to claim disability benefits at the Employee Insurance Agency (UWV), which is the Dutch social security agency. Besides of handling the benefits for employees, the UWV also regulates the sickness benefit for individuals that are not employed [16].

## Methods

### Study Design and Population

This study was conducted using data of Arbo Unie, a Dutch occupational health service. The total workforce of the Netherlands in 2021 was 9.66 million, of which 9.23 million had paid work [17]. Arbo Unie keeps track of data regarding sick leave of approximately 1.24 million Dutch workers from roughly 11.6 thousand companies of various sectors throughout the country [18]. Of these companies, about 82% have less than 200 employees, 17% have 200 to 10,000 employees and the rest have more than 10,000 employees. Data were obtained from workers who had seen an occupational physician and were absent from work at any time during the period of 2013 up to and including 2019. To minimize distortion of a sick leave episode’s duration, it was decided not to include the years 2014 and earlier as there was a possibility that an individual had started absenteeism before 2013. In addition, the decision was made not to include absenteeism that started in 2018 or later, as this could theoretically last beyond 2019. Furthermore, according to the Medical Ethical Committee of the VUmc in Amsterdam this study was not subject to the Medical Research Involving Human Subjects Act. Moreover, employees were included in this study if they fulfilled the following inclusion criteria: diagnosed with a stress related illness; age of 18 up to and including 65 years; contractual working week of at least 12 h and a maximum of 60 h; started sick leave within the period of 2015 up to and including 2017; ended sick leave within the period of 2015 up to and including 2019; and information on gender and the rate of absenteeism was available in the dataset. 

### Outcomes

Anonymized worker-level data were obtained from Arbo Unie. Data were obtained on the employees’ sex (male/female), age (years), diagnosis, and number of weekly working hours, the start and end date of absenteeism, and the rate of absenteeism (in %). Diagnosis and the estimation of the duration and cost of sick leave will be discussed into greater detail below:

#### Diagnosis

In the system of Arbo Unie, the employees’ diagnoses were classified according to a Dutch classification system, using CAS codes for occupational health services and social insurance institutions. This system is similar to the 'International Statistical Classification of Diseases and Related Health Problems' (ICD-10). For the purpose of this study, the following conditions were classified as stress-related illnesses: “tension complaints”, “overexertion”, “burn-out”, and “other reactions to stress” [19]. To be diagnosed with “tension complaints”, an individual must have at least three of the following symptoms: fatigue, irritability, disturbed sleep, worrying, forgetfulness, difficulty concentrating, agitated feeling, lability and difficulty to cope with stimuli or noise. To be diagnosed with “overexertion”, an individual must, in addition to tension complaints, also suffer from loss of control and significant limitations in social and/or professional functioning. In addition, these complaints may not be the sole result of another psychological disorder. If an “overexertion” lasted longer than six months, it was classified as a “burnout”. Individuals who did not meet all the diagnosis criteria for any of the previous disorders, but who did have complaints in this spectrum were classified under the category “other reactions to stress”. Furthermore, when during one episode of sick leave an employee was diagnosed with more than one condition, the most recent diagnosis was used, because it was assumed that the change in diagnosis was due to a physician having progressive insights.

#### Duration and Costs of Sick Leave

In accordance with the definition of UWV, a sick leave period was assumed to have a maximum duration of two years and is said to end after more than 28 consecutive days of full recovery [15]. First, for all employees, the number of sick leave periods and their associated number of calendar days was estimated based on the start and end dates of absenteeism. Then, these calendar days were converted into working days lost. This was done by dividing the number of calendar days by seven and multiplying that value by the number of working days per week (i.e. weekly working hours divided by 8.0), while correcting for (partial) resumption of work. The working days lost were valued using age-, gender- and year-specific wage rates [20, 21]. For estimating costs, we used three scenarios; (1) a scenario where it has been agreed in the collective labor agreement that 100% of the gross wages is reimbursed in the first year of absenteeism and 70% in the second year (i.e. base case scenario); (2) a scenario where the employer only fulfills their legal obligation and reimburses 70% of the employee’s gross salary during the entire absenteeism period (i.e. low-cost scenario); and (3) a scenario where it has been agreed to reimburse the full gross salary during the complete duration of absenteeism (i.e. high-costs scenario). The base-case scenario is assumed to occur most common [15].

### Analysis

Data were analyzed descriptively, using means and standard deviations for normally distributed data and medians and interquartile ranges for skewed data. We decided to report means for skewed data as well, because means are more informative for decision-makers than medians when absence durations and costs are involved. Analyses were performed for all three scenarios and stratified analyses were performed for gender. Data were analyzed using SPSS v27.

## Results

During the period of 2015 up and until 2017, a total of 16,676 employees started a sick leave episode due to stress-related illness. These employees had a total of 17,338 episodes of sick leave (Table 1), meaning that some of them experienced more than one period of sick leave during the study period. Most episodes of sick leave were attributed to “overexertion” (68.9%), followed by “tension complaints” (17.5%), “burn-out” (10.3%), and “other reactions to stress” (3.4%). Furthermore, women took sick leave due to stress-related illness more often than men (♀57.2%/♂42.8%). Women tended to suffer from “overexertion” more often than men (♀58.7%/♂41.3%), whereas this ratio was almost equal for men and women with “tension complaints” (♀51.0%/♂49.0%). The employees’ mean age at the start of a period of sick leave due to stress-related illness was 45.6 (SD = 10.7) years old. On average, in our sample women were younger than men (♀44.2 years/♂47.4 years). The mean number of contracted weekly working hours was 32.4 (SD = 7.4), and was found to be higher among men with 36.5 (SD = 4.8) hours a week than among women with 29.3 (SD = 7.5) hours a week.

**Table 1:** The number of episodes of sick leave, mean age at start of sick leave and the number of weekly contractual working hours stratified by different stress-related illnesses and gender

		Tension complaints	Overexertion	Burn-out	Other reactions to stress	Total
Number of periods of sick leave (%)	Female ♀	1545 (51.0%)	7009 (58.7%)	1036 (58.3%)	340 (57.5%)	9917 (57.2%)
	Male ♂	1485 (49.0%)	4931 (41.3%)	741 (41.7%)	251 (42.5%)	7421 (42.8%)
	Total	3030 (17.5%)	11,940 (68.9%)	1777 (10.3%)	591 (3.4%)	17,338 (100%)
Mean age in years (SD)	Female ♀	44.6 (10.9)	44.1 (10.6)	44.4 (10.2)	43.7 (10.7)	44.2 (10.6)
	Male ♂	47.7 (10.6)	47.3 (10.5)	47.9 (10.2)	47.5 (10.7)	47.4 (10.5)
	Total	46.1 (10.9)	45.4 (10.7)	45.8 (10.3)	45.3 (10.9)	45.6 (10.7)
Mean weekly working hours (standard deviation)	Female ♀	29.5 (7.8)	29.1 (7.4)	29.8 (7.1)	29.1 (7.5)	29.3 (7.5)
	Male ♂	36.6 (4.8)	36.5 (4.8)	37.0 (4.4)	35.9 (4.9)	36.5 (4.8)
	Total	33.0 (7.4)	32.2 (7.4)	32.8 (7.1)	32.0 (7.4)	32.4 (7.4)

On average, one episode of sick leave due to stress-related illnesses lasted 202 calendar days (Table 2), equaling 101 (♀102/♂100) working days. The duration of one episode of sick leave was highest for “burn-out” (163 days), followed by “overexertion” (101 days), “other reactions to stress” (100 days), and “tension complaints” (62 days).

**Table 2:** The mean duration in calendar days of an episode of sick leave due to stress-related illness and the lost working days in that period stratified to gender and accompanied by the median and interquartile ranges

		Tension complaints	Overexertion	Burn-out	Other reactions to stress	Total
Mean duration of sick leave in calendar days (Median; IQR)	Female ♀	134 (82; 148)	220 (174; 201)	329 (302; 264)	225 (180; 235)	218 (171; 217)
	Male ♂	109 (62; 124)	186 (147; 177)	291 (257; 214)	179 (132; 174)	181 (139; 187)
	Total	122 (70; 138)	206 (164; 190)	313 (280; 247)	206 (160; 206)	202 (158; 204)
Mean lost working days (Median, IQR)	Female ♀	62 (35; 65)	100 (74; 96)	153 (134; 135)	99 (72; 99)	100 (73; 101)
	Male ♂	62 (34; 66)	103 (78; 100)	171 (149; 132)	102 (78; 95)	102 (75; 107)
	Total	62 (34; 65)	101 (76; 98)	163 (139; 133)	100 (75; 99)	101 ( 74; 103)

In the base-case analysis, an episode of sick leave due to stress-related illness was found to cost Dutch employers €19,151 (Min. €13,867, Max. €19,811) in gross wages and slightly less for women than for men (♀ €17,248/♂ €21,700). The costs per sick leave period was highest for “burn-out”, followed by “overexertion”, “other reactions to stress”, and “tension complaints” (Table 3). An overview of the estimated average cost per sick leave period due to stress-related illness for the high- and low-cost scenario can be found in Table 3.

**Table 3:** The mean costs of gross salary for the employer of a period of sick leave for different scenarios, namely the base case scenario (payment of 100% of the gross salary in the first year and 70% in the second), the low-costs scenario (payment of 70% of the gross salary), and the high-costs scenario (payment of 100% of the gross salary) stratified by gender and accompanied by the interquartile ranges and stratified to the different stress-related illnesses

		Tension complaints	Overexertion	Burn-out	Other reactions to stress	Total
Base case scenario (€): 100% (1^ST^ year) and 70% (2^ND^ year) M (Median; IQR)	Female ♀	10,761 (6153; 11,715)	17,262 (13,113; 17,014)	26,840 (23,666; 23,129)	17,201 (12,880; 17,789)	17,248 (12,912; 18,100)
	Male ♂	13,189 (7288; 14,470)	22,082 (16,782; 22,458)	36,264 (31,712; 30,161)	21,575 (16,508; 22,501)	21,700 (15,882; 23,651)
	Total	11,952 (6670; 12,879)	19,254 (14,526; 19,426)	30,770 (26,814; 27,151)	19,058 (13,732; 19,989)	19,151 (14,032; 20,414)
Low-cost scenario (€): 70% only M (Median; IQR)	Female ♀	7731 (4307; 8217)	12,535 (9184; 12,049)	19,852 (16,795; 17,037)	12,496 (9016; 12,715)	12,550 (9038; 12,830)
	Male ♂	9445 (5102; 10,129)	15,863 (11,748; 15,779)	26,541 (22,199; 21,134)	15,541 (11,556; 15,751)	15,633 (11,124; 16,614)
	Total	8572 (4669; 9015)	13,910 (10,168; 13,598)	22,642 (18,770; 19,005)	13,789 (9612; 13,992)	13,867 (9828; 14,427)
High-costs scenario (€): 100% only M (Median; IQR)	Female ♀	11,044 (6153; 11,738)	17,906 (13,120; 17,213)	28,361 (23,993; 24,339)	17,851 (12,880; 18,163)	17,929 (12,912; 18,328)
	Male ♂	13,493 (72,88; 14,470)	22,662 (16,782; 22,542)	37,916 (31,712; 30,190)	22,201 (16,508; 22,501)	22,333 (15,891; 23,735)
	Total	12,245 (6670; 12,879)	19,872 (14,526; 19,426)	32,345 (26,814; 27,151)	19,699 (13,732; 19,989)	19,811 (14,039; 20,612)

## Discussion

### Main Findings

This study found that the average duration of a Dutch employee’s episode of sick leave due to stress-related illness was 202 calendar days, equaling a total number of working days of 101 days. Both the duration and costs of one episode of sick leave was found to be highest for “burn-out”, followed by “overexertion”, “other reactions to stress”, and “tension complaints”. In the base-case analysis, an episode of sick leave due to stress-related illness was found to cost Dutch employers on average €19,151 in gross wages and slightly less for women than for men (♀ €17,248/♂ €21,700). Naturally, in the low-cost scenario the costs were lower, at €13,867 (♀ €12,550/♂ €15,633) and in the high-cost scenario the costs were higher €19,811 (♀ €17,929/♂ €22,333). Please note that the difference between the base-case and the high-cost scenario is relatively small given that relatively few periods of sick leave last longer than a year.

### Comparison with the Literature

Comparing our results with literature is hampered by the fact that relatively few studies have assessed the duration of stress-related sick leave and that the corresponding costs for the employer, to the best of our knowledge, have not been examined yet. In the studies that did assess these costs partial resumption of work was not taken into account and different definitions of stress-related illnesses were used. Nonetheless, a Dutch study by Bakker et al. found a median duration of sick leave due to stress-related issues of 170 (143–197) calendar days, whereas we found a slightly lower median duration of 158 calendar days [22]. Another study by Roelen et al. found a median duration of sick leave of 109 (107–111) calendar days, but their study included sick leave due to stress-related illness as well as neurotic and somatoform disorders [23]. A press release from a Dutch occupational health service stated that they found sick leave due to “burn-out” to last on average 242 calendar days, whereas sick leave due to “tension complaints” had an average duration of 167 calendar days [24]. We found a longer sick leave duration for “burn-out” (on average 313 calendar days) and a shorter sick leave duration for “tension complaints” (on average 122 calendar days). However, the methods and classification of stress complaints were not revealed by the press release, while differences in methods and classifications could explain the differences between findings. A handful of studies has also assessed the duration of sick leave due to stress-related illness in other countries (e.g. Sweden [11, 12], Finland [13]), but comparing our results with those from other countries is hampered by differences between countries in culture, insurance systems, and diagnostic classification systems, all of which could influence the duration, and thus the costs, of sick leave [25, 26]. For instance, two Swedish studies, based on data of the National Social Insurance registers, found durations of sick leave due to stress-related illness of respectively 49 and 55 calendar days per episode [11, 12]. These durations are considerably lower than our finding of 158 calendar days. This difference can largely be explained by the fact that in Sweden the payment obligation for the employer is only two weeks, after which the employee is admitted to the Swedish sick insurance system and receives benefits [27]. As a result, relatively short-term sick leave periods were likely registered in their study, whereas in our study mainly absenteeism that lasts longer than six weeks is registered.

We also found women to have longer sick leave durations due to stress-related illness than men. This might be related to the fact that women who suffer from mental health problems are more likely to seek help from a doctor, other practitioners, and/or friends than men [28]. It is conceivable that by seeking help women initiate the process of sick leave earlier, while men continue working whilst ignoring their symptoms. It is important to mention that this does not necessarily mean that men have lower total productivity-related costs than women. Continuing to work while experiencing health complaints could lead to lower productivity levels while present at work as well, also known as presenteeism. As we did not have information on presenteeism available in our dataset, further research is needed to establish this. Another explanation could be related to the professions of men and women. It is known that women and men tend to work in different sectors [29]. Women, for instance, work more often in the educational and healthcare sector, which are known to have relatively high job and emotional demands and low job control, all of which are predictors for sick leave [30–32]. Emslie et al. even hypothesized that differences in sick leave would not exist if men and women would have the same profession under the same circumstances [33]. Of interest was that women had longer sick leave durations due to stress-related illness than men in terms of calendar days, whereas the number of working days lost due to stress-related illness was the same for men and women. Our results thus support the hypothesis of Emslie et al. given that women on average work fewer hours per week but still have about the same number of lost working days. Future research might assess the underlying cultural and social causes of the differences between men and women with regard to sick leave due to stress-related illness, which could cover a wide range of aspects, such as: ‘Do women's sick leave relate to having children?’ to ‘Do occupational physicians focus more on getting the breadwinner of the family back to work?’. Moreover, despite women having about the same number of lost working days due to stress-related illness as men, the costs per episode of sick leave for men were higher than for women. The difference is caused by the pay gap between men and women and the fact that women are on average younger than men at the start of an episode of sick leave due to stress-related illness [20, 21].

### Strengths and Limitations

Multiple aspects of this study contributed to its strength. The large customer base of Arbo Unie, in combination with the obligation for the employer to contact an occupational health service during the employee’s sick leave provided us the opportunity to assess the duration and costs of sick leave due to stress-related illness using a large and representative sample of more than 16.6 thousand employees with diverse professions in all layers of society. This likely increased the generalizability of our findings to Dutch workers in general. Furthermore, many studies have faced problems with recall bias due to a dependency on self-reported dates of sick leave [34–36], whereas our study was not affected by recall bias since dates of sick leave were meticulously reported by occupational physicians and maintained by Arbo Unie in their databases. Furthermore, the availability of recovery percentages allowed us to estimate the number of lost working days corrected for partial work resumption, which to the best of our knowledge has never been determined before. This is important for a precise estimation of the lost working days and therefore the costs which employers are in need of to prepare for absenteeism and shield themselves from possible high related costs when for example considering whether to take out sick leave insurance or not [10].

This study, however, is also subjected to some limitations. First of all, an employee is obliged to visit an occupational physician during the first six weeks after reporting in sick for the first time. The dataset of the current study is based on information of occupational physicians and thus includes absences of employees who visited their occupational physician within the legislated six-week period and reported better again in or after this period, or became permanently unable to work. Employees who report sick to their employer but recovered before seeing an occupational physician are not included in this dataset. It is unclear to what extent this occurs, however it is clear that this could have led to an overestimation of the average duration and cost per period of sick leave. This overestimation will mainly apply to complaints of a shorter nature, such as "tension complaints" and "other reactions to stress", but the exact magnitude of this overestimation remains unknown. Moreover, it is well known that mental disorders have high comorbidity rates with other mental as well as somatic disorders [37, 38]. Therefore, the estimated duration and cost of sick leave due to stress-related illnesses could be distorted by imperceptible influences of other disorders. Lastly, it should be noted that presenteeism, i.e. reduced productivity due to illness while an individual is present at work, were not included in our study. Consequently, the total cost of productivity losses due stress-related illness, which consists of both absenteeism and presenteeism costs, is higher than the estimates of our study [39].

### Recommendations for Practice

It was already known that a large proportion of sick leave is caused by mental disorders, including stress-related complaints. As other studies have also found, the duration of sick leave due to stress-related illness is high, which results in substantial costs [22–24]. In total, the average annual number of employees starting a period of sick leave due to stress-related complaints would cost approximately €799 million, if we were to make a rough estimate taking into account the working population in the relevant starting years and assuming that the number of Arbo Unie customers remained the same [17]. This is a substantial part of the total estimated annual salary payment due to illness in the Netherlands of 11.5 billion euros [40]. Obviously, not every (Dutch) organization can afford more than 19 thousand euros when an employee turns ill. Furthermore, sick leave has many other negative side effects, such as an increased workload, loss of valuable knowledge and extra costs for replacement staff. Therefore, an appropriate approach must be considered to alleviate the burden on the employer. A solution to reduce this burden could be sought in research and implementation of preventive measures together with adequate interventions to keep the number of days of sick leave and therefore the costs as low as possible. Various studies have already shown that such measures can have a beneficial effect on the course of stress-related illness and its sick leave [41–43]. Another option may be to reduce the compensation for sick leave due to illness. Research shows that the more abundant the compensation is for sick leave, the more days employees are absent [44]. To a certain extent this explains why a relatively high absenteeism rate is found in countries such as the Netherlands, Sweden and Norway, whereas countries with a shorter period of paid sick leave such as the United Kingdom, Italy and Switzerland have relatively little sick leave [45]. However, the fact that absenteeism and presenteeism are inextricably linked should also be taken into account. Research shows that measures to reduce absenteeism can result in employees returning to work whilst still partially sick and not fully productive (i.e. presenteeism) [46, 47]. The costs of presenteeism can even far exceed those of absenteeism [48, 49]. Another possible solution to lighten the employers’ burden, which might be worth investigating further, is a form of compulsory sick leave insurance for all Dutch companies. The demand for sick leave insurance in the Netherlands is already high, especially among small businesses where about 80% is insured [50]. A compulsory sick leave insurance for companies with an element of the 'ability to pay principle' could maybe lower premiums and make sick leave affordable for every employer.

## Conclusion

It is important for employers to keep employees in good mental health so that they do not fall ill. The results of this study indicated that the duration and costs of sick leave due to stress-related illness are substantial, especially for “burn-out”. This study will hopefully not only act as an eye-opener for employers but will also provide insights to adequately prepare for sick leave due to stress-related illness (e.g. when considering whether to take out insurance or not). Approaches should be sought and/or implemented to promote mental well-being and alleviate the financial burden of sick leave due to stress-related illness on the employer, such as implementation of preventive measures and adequate interventions.

## Data Availability

Data is only visible on site to editors on request due to business sensitivity.

## Code Availability

SPSS codes are available on request.
